# Assets for integrating task-sharing strategies for hypertension within HIV clinics: Stakeholder’s perspectives using the PEN-3 cultural model

**DOI:** 10.1371/journal.pone.0294595

**Published:** 2024-01-02

**Authors:** Juliet Iwelunmor, Ifeoma Maureen Obionu, Gabriel Shedul, Ekanem Anyiekere, Daniel Henry, Angela Aifah, Chisom Obiezu-Umeh, Ucheoma Nwaozuru, Deborah Onakomaiya, Ashlin Rakhra, Shivani Mishra, Erinn M. Hade, Nafesa Kanneh, Daphne Lew, Geetha P. Bansal, Gbenga Ogedegbe, Dike Ojji

**Affiliations:** 1 Department of Behavioral Science and Health Education, College for Public Health and Social Justice Saint Louis University, St. Louis, MO, United States of America; 2 Department of Family Medicine, University of Abuja Teaching Hospital, Gwagwalada, Abuja, Nigeria; 3 Cardiovascular Research Unit, University of Abuja Teaching Hospital, University of Abuja, Gwagwalada, Abuja, Nigeria; 4 Department of Community Medicine, Faculty of Clinical Sciences, University of Uyo, Uyo, Nigeria; 5 Department of Population Health, New York University Grossman School of Medicine, New York, New York, United States of America; 6 Department of Implementation Science, Wake Forest School of Medicine, Winston-Salem, North Carolina, United States of America; 7 Washington University in St. Louis School of Medicine, St. Louis, MO, United States of America; 8 Fogarty International Center, NIH, Bethesda, MD, United States of America; 9 Institute for Excellence in Health Equity, New York University Grossman School of Medicine, New York, New York, United States of America; 10 Department of Internal Medicine, Faculty of Clinical Sciences, College of Health Sciences, University of Abuja, Gwagwalada, Abuja, Nigeria; University of Ghana School of Pharmacy, GHANA

## Abstract

**Background:**

Access to antiretroviral therapy has increased life expectancy and survival among people living with HIV (PLWH) in African countries like Nigeria. Unfortunately, non-communicable diseases such as cardiovascular diseases are on the rise as important drivers of morbidity and mortality rates among this group. The aim of this study was to explore the perspectives of key stakeholders in Nigeria on the integration of evidence-based task-sharing strategies for hypertension care (TASSH) within existing HIV clinics in Nigeria.

**Methods:**

Stakeholders representing PLWH, patient advocates, health care professionals (i.e. community health nurses, physicians and chief medical officers), as well as policymakers, completed in-depth qualitative interviews. Stakeholders were asked to discuss facilitators and barriers likely to influence the integration of TASSH within HIV clinics in Akwa Ibom, Nigeria. The interviews were transcribed, keywords and phrases were coded using the PEN-3 cultural model as a guide. Framework thematic analysis guided by the PEN-3 cultural model was used to identify emergent themes.

**Results:**

Twenty-four stakeholders participated in the interviews. Analysis of the transcribed data using the PEN-3 cultural model as a guide yielded three emergent themes as assets for the integration of TASSH in existing HIV clinics. The themes identified are: 1) extending continuity of care among PLWH; 2) empowering health care professionals and 3) enhancing existing workflow, staff motivation, and stakeholder advocacy to strengthen the capacity of HIV clinics to integrate TASSH.

**Conclusion:**

These findings advance the field by providing key stakeholders with knowledge of assets within HIV clinics that can be harnessed to enhance the integration of TASSH for PLWH in Nigeria. Future studies should evaluate the effect of these assets on the implementation of TASSH within HIV clinics as well as their effect on patient-level outcomes over time.

## Background

While HIV remains a leading cause of premature death among adults in Africa, the burden of non-communicable diseases such as hypertension is increasing rapidly [1–3]. Currently, in Nigeria, Africa’s most populous country, representing nearly 9% of the estimated burden of people living with HIV in Africa, HIV, and hypertension comorbidity are on the rise [4, 5]. Access to anti-retroviral therapy has improved survivorship among PLWH while simultaneously expanding the number of PLWH in Africa [6, 7]. As a result, the prevalence of hypertension among PLWH in Nigeria is estimated to range from 24–34% [8–10]. Throughout Africa, approaches to achieve optimal management and control of hypertension remains inaccessible for PLWH [11, 12]. Barriers at the individual (limited patient knowledge of hypertension), provider (lack of training), health systems (shortage of staff, limited medications, and equipment), and policy level (poor uptake of policy recommendations) levels limit efforts to maximize the benefits of optimal hypertension management and control among PLWH [12, 13] Furthermore, most existing HIV services are not designed to provide continuity of care, including identifying PLWH with increased risk of hypertension, linking them to effective care and retaining them over time in care [1, 14–16]. This has led to fragmented services, thus highlighting the need to improve patient health by optimizing the use of evidence-based hypertension care for PLWH in Africa.

Task sharing or the distribution of some clinical services from physicians to non-physician specialists is an evidence-based implementation strategy that may mitigate some of the barriers to the integration of hypertension care within routine HIV services in Nigeria [17–19]. Due to the benefits of task sharing, Nigeria established a task-sharing and sharing policy in 2014 with the goal to promote access to health services while efficiently utilizing other cadres of healthcare workers such as nurses and community health workers in routine settings [7]. Additional benefits include bridging the gap caused by the shortage of physicians and poor access to services, limiting Nigeria’s capacity to manage hypertension in primary healthcare facilities where most PLWH receive care [7, 20]. Task-sharing strategies for hypertension care (TASSH) is based on the WHO Package of Essential Non-Communicable Disease Interventions and it includes CVD risk assessment, medication titration, lifestyle counseling, and patient referral [18]. TASSH has been successfully implemented among a general population of hypertensive patients in 32 community health centers in Ghana and led to 34% greater systolic blood pressure reduction among patients with the intervention versus those with health insurance coverage alone [19]. The National AIDS Control Agency in Nigeria also recommends the integration of care for hypertension within existing HIV services for the dual control of hypertension and HIV [7]. Several potential benefits of the HIV-NCD integration within existing HIV services include: a decrease in the duplication and division of health services provided, an increase in the efficiency of the use of resources especially in low-limited resource settings, increased promotion of patients retention in care, and reduction in costs and inconvenience for patients living with multiple illnesses [21, 22]. For PLWH, promoting early identification and management of NCDs within the HIV care programs may lead to a reduction in morbidity and mortality [15]. Despitethese benefits and guidelines, including the recommended use of task-sharing strategies for hypertension care, integration of these strategies within HIV services remains suboptimal [21–23].

Stakeholder engagement can provide insight into specific implementation factors likely to influence the integration and adoption of evidence-based task-sharing strategies for hypertension control within HIV services [12, 24–27]. Not only are they key decision-makers with implementation, but their opinions may influence whether evidence-based task-sharing strategies for hypertension care are implemented or adopted within HIV services [27]. Prior studies suggest that stakeholder engagement during the implementation process may help to identify factors that may influence implementation, optimize care delivery strategies, and uncover readiness and/or apprehension around change, while providing training, and performance feedback to change recipients of the interventions [1]. Stakeholders can expedite the implementation process, by highlighting relevant and innovative perspectives that are most efficient and may effectively move research to practice in their own settings [28]. Limited attention to stakeholders’ perspectives may lead to failed integration and adoption of evidence-based task-sharing strategies for hypertension control [24]. Despite their significance, stakeholder engagement in the integration of evidence-based task-sharing strategies for hypertension control remains incompletely understood and limited especially in Africa.

**Fig 1:**
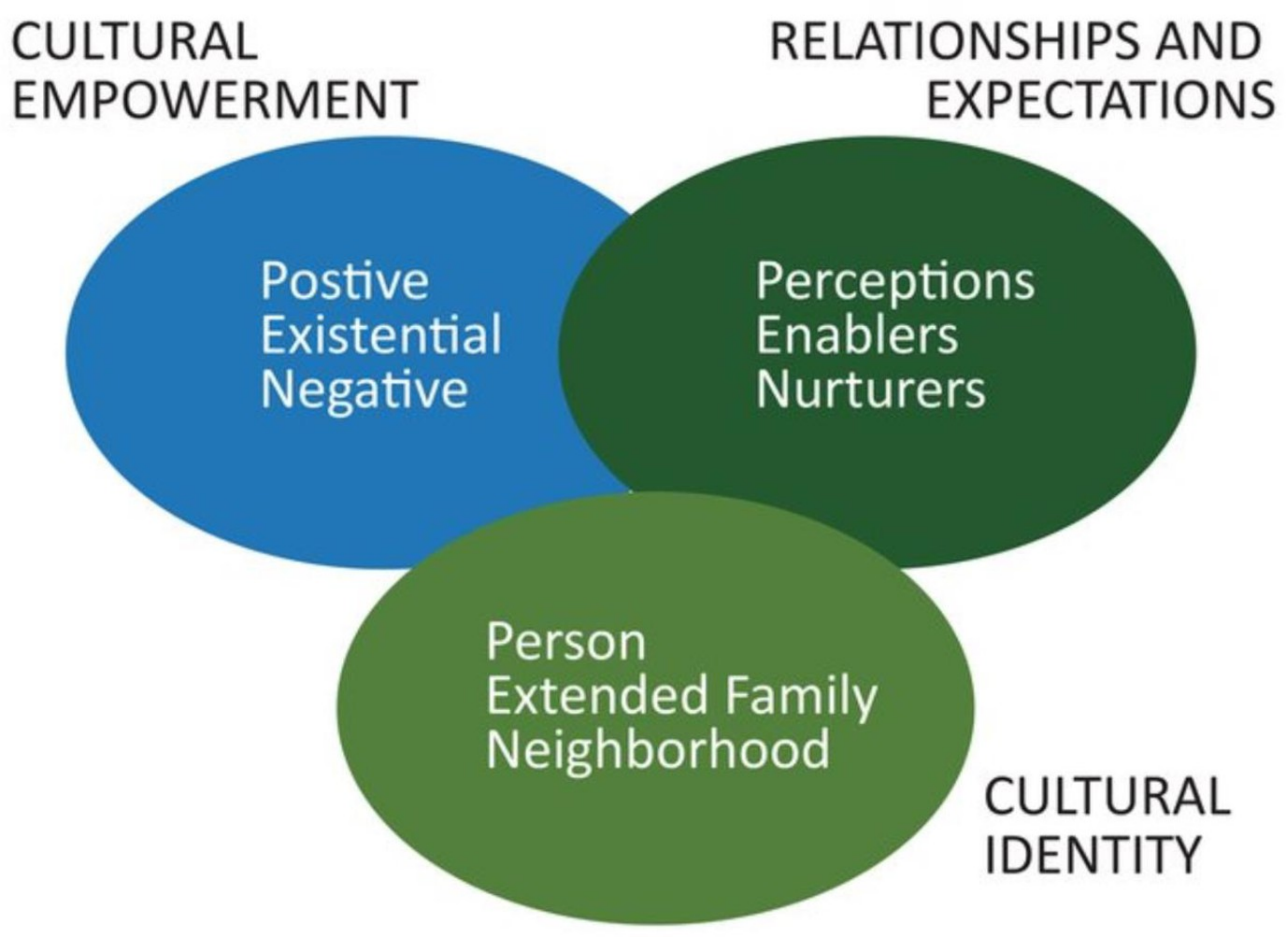
The PEN-3 cultural model [31].

The need for more research-informed knowledge on assets (health-promoting factors operating at different levels within health systems) that can be leveraged for health interventions was recognized decades ago, yet the issue did not receive high visibility [29]. Recently, a report on efforts to eliminate persistent health disparities, has called for researchers to move away from a deficit mindset of what individuals and communities are doing poorly (i.e., barriers to implementation) to one that is positive about what they can collectively achieve [30]. Furthermore, evidence-based interventions are more likely to be implemented in systems where existing assets, consistent with the local contexts, are harnessed over time. We define assets here as positive or unique factors within health systems that can be leveraged to enhance the implementation of evidence-based interventions in resource-limited settings [31]. Assets are rarely considered when planning interventions in many resource-limited settings. Yet, an asset-based approach to health can help increase the resourcefulness of these settings to improve their health, while maintaining and sustaining health benefits over time to reduce health inequities [29]. As a result, we used the PEN-3 cultural model (Fig 1), an asset-based framework that recognizes the capacities and capabilities of individuals and their communities to promote health, as a guide [31]. The PEN-3 cultural model can help to identify contextual and culturally compelling factors likely to influence implementation processes in resource-limited settings from a strength-based perspective and not solely narratives of deficit [32–34]. Historically, implementation science research conducted in African settings used frameworks that fail to account for the cultural context in which health issues are addressed [35–39]. As a result, the role culture may play in implementation, adoption, and or sustainability are often invisible and thus overlooked, while implementation strategies used, fail to achieve any impact on health interventions over time [40, 41]. The PEN-3 cultural model is a flexible model that enables researchers to address this gap. This model consists of three domains: Cultural Identity (Positive, Existential, and Negative), Relationships and expectations (Perceptions, Enablers, and, Nuturers), and Cultural empowerment(Person, extended family, and Neighbourhood) [31]. These domains can be used to identify not only relationships and expectations or perceptions, enablers or resources and nurturers or what to continue with a particular implementation project, but also cultural empowerment aspects of the project or factors that are positive, existential or unique as well as those that are negative and may impact the implementation process [39–44]. PEN-3 acknowledges the complexity of implementation in settings limited with resources, incorporating a broader view of not only barriers to implementation but factors within the settings that are unique and may be viewed as assets towards the implementation process [37]. This paper presents a detailed assessment of stakeholders’ perspectives on efforts to integrate evidence-based task-sharing strategies for hypertension care within HIV services in Nigeria. Findings may provide a better understanding and thus advance the preparation of contextually compelling assets for enhancing the adoption of task-sharing strategies for hypertension among PLWH in Nigeria. Additionally, we provide insight into using the PEN-3 cultural model and comment on its utility with implementation science research conducted in African settings.

## Methods

### Participants and setting

A purposive sample of key stakeholders, including PLWH patient advocates, community nurses, chief medical officers, and policymakers from HIV services in Akwa Ibom, Nigeria, were identified and recruited through face to face method by the research team to participate in in-depth interviews. Akwa Ibom is a southern state in Nigeria with one of the highest prevalences of HIV in Nigeria [45]. It is one of four states in the South-South and South-East region of Nigeria with a relatively high HIV prevalence of 2% or above [45]. It is also among key priority states that account for over 50% of PLWH in Nigeria, new HIV infections, and death from HIV [45].

Twenty-four key stakeholders were interviewed and included: six PLWH who also served as patient advocates, ten health care providers consisting of six nurses and four medical officers, and five policymakers and three directors of primary health care clinics in Akwa Ibom. Participants were between 34 to 60 years of age and the majority (71%) were female (Table 1).

**Table 1:** Sociodemographic characteristics of respondents.

	Frequency (n)	Percentage (%)
**Occupation**		
Nurse	6	25.0
Medical officer	4	16.7
Policy maker	5	20.8
Director of Primary Health Clinic	3	12.5
Patient Advocate	6	25.0
**Gender**		
Male	7	29.3
Female	17	70.8
**Age (years)**		
34–50	8	33.3
Greater than 50	16	66.7
**Educational level**		
Bachelors	17	70.8
Postgraduate	7	29.2
**Years of work experience (Health care professionals)**		
10–20	5	27.8
21–30	6	33.3
31–40	7	38.8

### Study design

This study is part of a larger implementation trial aiming to investigate the integration of an evidence-based task-sharing strategy for the management of hypertension (TASSH) within HIV clinics in Nigeria. In the larger implementation trial we adapted TASSH to include treatment of uncomplicated hypertension by Community Nurses using the Nigeria Hypertension Treatment Protocol [46]. The present study used semi-structured in-depth interviews with key stakeholders involved with HIV service delivery at clinics in Akwa Ibom State of Nigeria. This approach was chosen because it offers a deeper understanding of stakeholder perspectives, including lived experiences with providing health care services for PLWH that may impact the integration of evidence-based task-sharing strategies for hypertension within existing HIV services [44].

### Data collection

We developed an interview topic guide that focused on factors likely to influence the integration and adoption of TASSH within HIV clinics in Akwa Ibom, Nigeria such as the relative advantage of TASSH, alignment with existing clinic structures, patient support, and alignment with national guidelines, the perceived complexity of TASSH, and limited resources [1]. We piloted the draft interview guide for comprehensibility, flow, and meaning, with two individuals who met all inclusion criteria and were not participating in the study. The interview guide was modified based on feedback prior to use in the field. 2 experienced researchers who both public health professionals conducted the interviews with key stakeholders. Data collection took place from May 31^st^ to June 11^th^ 2021. Only the interviewer and interviewee were present during the interviews. Specifically, they asked participants to first describe their perceptions of the TASSH project, its potential impact on the workflow of staff as well as current patient experience and clinical encounters within HIV clinics. Ethical approval was obtained from The University of Abuja, Nigeria institutional review board (Approval number UATH/HREC/PR/2020/006). Written consent was obtained from all participants consistent with the procedures approved by the University of Abuja, Nigeria institutional review board prior to the interviews. Interviews were carried out until saturation. The Interviews lasted for one hour and were audio recorded. Not identifiable information was obtained from participants.

### Data analysis

The interviews were transcribed verbatim and uploaded into Microsoft Excel for coding and thematic analysis by 2 trained research assistants. We applied a two-prong approach: categorizing coded data using the PEN-3 cultural model and analyzing the interviews using the Framework Approach for qualitative analysis [47]. The Framework Approach is a systematic process in which case-level data (rows) are summarized along thematic categories (columns) in a matrix. It involves five steps that include: 1) familiarization with the data: 2) identifying a thematic framework: 3) indexing; 4) charting, and 5) mapping and interpreting the data [47]. We structured the coding process using the PEN-3 cultural empowerment domain of positive, existential, and negative factors as well as the relationship and expectation domain of perceptions, enablers, and nurturers like to impact the integration of TASSH within existing HIV serves in Akwa Ibom [43]. Coding began with an independent reading of the transcripts by the 2 research assistants to compare coding, codes and discuss emergent themes. Following agreement on definitions and interpretation of codes, a coding strategy was developed and used to code the remainder of the interviews. Coded data were initially categorized using the PEN-3 domains, then, data were summarized, compared, and contrasted, until identifying patterns and themes emerged. To ensure rigor with the coding process, an audit trail of coding decisions were recorded and reviewed during meetings with the research team [48]. Key stakeholder quotes were also collected to provide thick description of their perspectives. To foster credibility, we linked data to categories in the PEN-3 cultural model to flesh out our understanding of the essential contextual and/or culturally compelling factors that may influence the integration of TASSH within HIV clinics in Nigeria. Standards for reporting qualitative research were used to guide the reporting of the data.

## Results

Through exploration of the PEN-3 cultural model domains of relationship and expectation (i.e. perceptions, enablers, and nurturers) as well as the cultural empowerment domain (positive, existential, and negative), we identified three key themes that are important assets for facilitating the integration of TASSH within HIV services in Akwa Ibom, Nigeria. We grouped these themes according to stakeholder participation as follows:

### Extending continuity of care for PLWH in familiar or comprehensive settings (patient advocates)

Some patient advocates believed that the implementation of TASSH would promote continuity of care for PLWH. Some suggested that it would make communication easier between patients and their providers. They also felt that it would be more helpful to have the same healthcare worker who is managing their HIV treatment also manage them if they have other illnesses, such as hypertension. One patient advocate stated the following:

*"I think it will be cool*. *Because from there*, *the people have already known your history*, *and they know the kind of drugs*, *they know the type of ART you are taking*, *and whether you are faced with high blood pressure and you will feel very free*, *because someone that you have been meeting*, *you have been meeting over years*, *and you will feel free to*, *actually say*, *this is what is happening to me*, *this is how it is*, *and the person knows your history already*. *So*, *he will know how to address other related issues*.*" PA06*

Another patient advocate noted that vital signs, including blood pressure readings, are ongoing as part of HIV service delivery in many clinics. However, having dedicated support would enhance the care being provided to PLWH, particularly with referrals to additional care. They noted the following:

*“Once the vital signs are being conducted when you arrive and your blood pressure is continually higher than a hundred and twenty, definitely that’s an indication that hypertension is around the corner, so, they (nurses at the health center) will refer you to further care in comprehensive sites*.*” PA-04*

Another stated that extending the continuity of care for PLWH within existing HIV services helps to address issues related to stigma. They shared the following:

*“It also helps to control stigma and is more confidential to the client if they continue to access their care in one place*, *because leaving here and going to another place*, *people will ask questions*. *They can say things like*, *“this one is having double chronic diseases”*. *Stigma will set in*.*” PA-05*

One stated it furthers ongoing interactions and communications between PLWH and their provider. They shared the following:

*“You will feel very free*, *because someone that you have been meeting*, *over the years*, *is the same person that you can actually say*, *this is what is happening to me*, *this is how it is*, *and the person knows your history already*. *So*, *they will know how to address other related health issues*.*” PA-06*

However, in cases when care is complex, one patient advocate stated that trained nurses often refer cases to comprehensive sites given the persistent problem of hypertension. They stated the following:

*“We (patient advocate) make a referral also from the primary health to comprehensive site*, *because they (nurses at the site) have been trained and have the ability to carry out new care given the ongoing hypertension problem in the state*.*” PA-04*

### Empowering health care workers (health care providers)

One of the key roles of healthcare workers is to meet PLWH needs across different chronic care contexts. As a result, some healthcare workers suggested there were more benefits than barriers to integrating hypertension care within existing HIV services. One shared the following:

*“I’m seeing much benefits than barriers or challenges*. *Benefit number one*, *the nurses in the community*, *they are the people that have a good rapport with PLWH*. *Thus*, *they are close to the patient and we have the techniques of ensuring that each visits the hospital… We are the one to check on them when they visit… I’m believing that when the workers will be trained from all cadres of nursing and the equipment made available*, *the job (TASSH) will be successful*.*" HCP04*

Additionally, healthcare workers stated that the measurement of patients’ blood pressure was a routine practice at most departments at their facilities and was convinced that the focus on strengthening the use of this practice will help in the integration of hypertension within routine HIV services at the clinics. One participant shared the following:

*"My primary health clinic is able to implement task-sharing because it is something we already do with the issue of blood pressure…we already measure this*. *It is a practice that has already been on the ground in several units*, *like the Maternity Unit*, *the Family Planning*, *and even the outpatient department*. *Now*, *incorporating it into the system will not be a problem*.*” HCP04*

One healthcare provider noted that task-sharing strategies for hypertension will enhance patient care, particularly in cases where patients need referrals or further treatment, rather than sending them home with no additional care. They stated the following:

*"we’ll gain much from it because instead of sending patients home*, *at least people will be there to take care of them*. *If the doctors are not there*, *at least the nurses or other workers can try to provide care*.*" HCP03*

Another who worked in a primary health clinic stated that these services (hypertension care) were not currently part of their duties to monitor overtime in their patients. However, because the cases are growing, such a program would empower them to provide routine screening and management among PLWH. They stated the following:

*“The benefits of task-sharing strategies are to prevent hypertension and also to build the capacity of the nurses and the CHEWs involved in the care*. *It will also help in the early diagnosis and*, *the early detection and diagnosis of hypertension as well as prevent complications with hypertension like stroke*, *kidney disease*, *heart failure*, *and sudden death*. *Currently*, *it is not the duty of nurses (at primary health centers) to provide hypertension care*, *however*, *we now have the opportunity (through such programs like TASSH) of being trained to manage PLWH with hypertension*.*” HCP06*

However in settings where these services are currently being provided, another health care worker noted that it would help to reduce the challenge of shortage of staff, but ensuring that other cadres of health care workers provide hypertension care, thus fostering teamwork between staff. They stated the following:

*“At this facility*, *the nurses are very few*, *very few nurses; and we divide them into shifts*. *By the time you put them into shifts*, *some shifts may result in no nurse at all…Because of that*, *we also try to shift some duties to the Community Health Extension Workers (CHEWs) that are with us*, *and I think that is the aspect of the task-sharing strategies that would be beneficial to us*, *so that they (CHEWs) can also be able to take blood*, *measure blood pressure and then record accordingly…because we don’t have enough nurses to do the work*. *But we have few nurses here*, *we have CHEWs here*, *we have health attendants*, *and all other workers*. *And I think in the issue of task shifting and sharing*, *everyone should be involved because it’s a team work*.*” HCP07*

Similarly, training and re-training of staff were recommended as one of the key strategies necessary to enhance the integration of hypertension care within routine HIV services in the country. One participant shared the following:

*“what will enhance the implementation of task-sharing strategies for hypertension care is having more staff that are trained and have the capacity to handle the program… when patients appear before them*, *and the card of these patients is before them*, *and since they are being incorporated in the TASSH program*, *they will now see the question on patient’s care*, *“Has this person’s blood pressure been checked*?*” If it’s not checked*, *okay*, *let me know the patient’s blood pressure*. *So*, *it will help*. *And if they discover the patient is hypertensive*, *they will make sure that the patient gets immediate care… as a result*, *there should be proper training and re-training of staff*.*” HCP06*

Access and availability of resources, including blood pressure monitoring equipment and the supportive monitoring of these resources, was suggested as another strategy necessary to address the leaky cascade of hypertension care among PLWH. These resources help to optimize the hypertension cascade for PLWH by ensuring they get proper care with screening, diagnosis, management, and control of their hypertension. One healthcare provider shared the following:

*“The first important thing is we must have a good blood pressure monitor*. *Because without having a monitor*, *you would not know when the blood pressure is high…Given the necessary equipment…; the necessary tools*, *and the necessary update of knowledge*, *they (staff) would be able to carry out the components of the TASSH program*.*” HCP07*

Alongside resources, is the need for collaboration and open communication among all focal persons within the existing HIV services. Participants believed that meetings with focal persons, held monthly in some settings or every two months in other settings were pivotal for adopting and sustaining new programs within their health systems.

*“Like in this facility*, *we have heads of units’ meeting; the focal persons would meet and*, *because we work as a team*, *you cannot just say I am doing family planning*, *and then*, *you-*, *you-*, *family planning cannot stand alone*, *immunization cannot stand*. *So*, *all the focal persons*, *we meet*, *and then we interact and make sure the team moves together*. *And*, *if you don’t do that*, *you know*, *somebody will stand alone and the program will not work…We have open dialog during these meetings…Because you know*, *without communication*, *you cannot succeed*.*”*

### Enhancing existing workflow, staff motivation, and stakeholder engagement (policymakers)

Among some policymakers, task-sharing strategies which are in alignment with existing policies will improve the efficiency of services at the primary healthcare level. Due to the shortage of doctors at this level of care, it is important that the few doctors available are managing the critical patients and some tasks such as hypertension screening can be handled by other healthcare workers thus promoting efficiency.

*“It (task shifting) will lessen burdens on the professional physicians and it will also make them more efficient*. *They will also have time to concentrate on very critical cases*. *It will also reduce the time patients will spend in the hospital”*. *PM03*

The implementation of TASSH was identified as a means of improving the capacity of healthcare workers in the primary healthcare system. Some policymakers believed that this will motivate healthcare workers to promote the uptake of TASSH in their facilities because it will be helpful in their skill development

“One, because it will empower the nurses and other health workers on how to manage the hypertensive cases, how to prevent other diseases such as the cardiovascular diseases” PM02

Staff motivation was identified by some policymakers as a way to facilitate the uptake of TASSH. They highlighted that it could be financial or non-financial, and could be as simple as the continuous appreciation of the effort of these healthcare providers involved in the TASSH process which will, in turn, encourage the healthcare providers to continue to implement TASH leading to the success of the program.

*“You need to also factor in how to motivate them*. *When we’re talking about motivating them*, *it might not just be financial motivation*. *No*. *From time to time*, *appreciate them and tell them*, *“O*, *you are useful*. *You are relevant*. *You’re contributing to the success of these things*. *Everybody*, *I think at that level*, *people want to be appreciated”*. *PM03*

Some policymakers also mentioned that in their facilities they had a culture of collaboration between all cadres of healthcare workers and a culture of open communication. They stated that this culture would be vital in promoting the implementation of task-sharing strategies for hypertension care. They explained that they had a routine team meeting where they discussed the best practices and how things can be done to benefit their patients. Such meetings are important for sharing updates on new programs.

*“Well*, *we collaborate actions*, *because we have monthly meetings where we review our activities*. *Once a month*, *we pick a particular case*, *discuss it among ourselves and we suggest the way forward*, *and what should be done*. *That is how we collaborate within ourselves”*.*PM01*

One policymaker noted that due to the high number of patients attending the primary care centers, limited staff, and other pertinent issues such as providing COVID-19 vaccinations and routine immunization services, limited attention is given to the identification and management of hypertension in patients within existing HIV services. These competing priorities should be considered when implementing new programs like TASSH as noted below:

*“We have a shortfall in the number of healthcare workers in the state*, *and so many programs are coming in at the same time*. *We are doing COVID-19 vaccination right now*. *Then*, *immunization plus days are also coming in*. *Net replacement campaign is also coming in*. *A lot of things” PM02*

Another policymaker noted that service provision was fragmented in some primary health centers with limited infrastructure, staff strength, and staff capacity across the region. As a result, all the PHCs may not be able to implement TASSH as intended. *The context in which the TASSH program will be implemented should be taken into consideration*. They shared the following:

*"The operational base*, *that controls the other smaller PHCs under them*, *might have better facilities*, *might have well-trained nurses*, *and some of those cases*. *So*, *you need to carefully select*, *you know*, *the facilities*, *you need to carefully select based on where you have the right infrastructure for you to work with*.*" PM03*

Finally, one policymaker shared that to facilitate the uptake of TASSH program, advocacy with all key stakeholders was important. They emphasized that this should include the health workers expected to implement the program, managers, and directors at the PHC facilities, service users, the community, the government, and policymakers. Also that the government and policymakers should ensure that there is an enabling environment for this program to be successfully integrated at the PHC level and a strategic health plan that includes this program.

*“The stakeholders would definitely have to include all people expected to be involved*. *So that’s the level of the government*, *from the community*, *people that are running the program*. *The state government provides the enabling environment*, *allowing this program to be fully incorporated formally as part of the things that are offered on a routine basis*. *Okay*, *So they advocate that level*. *Policy making level can be incorporated cause of this*, *just so that they provide the enabling environments and* a *Strategic health Plan will be needed for reflecting to what extent this program fits with priorities*.*” PM04*

## Discussion

Approaches to maximize the benefits of hypertension care for PLWH are needed in African countries like Nigeria, given the rising prevalence of hypertension [39]. Task-sharing strategies for hypertension care are evidence-based strategies that may be effective for incorporating hypertension diagnosis and management within existing HIV services in Nigeria [17]. However, integration of such services is a complex process that will require a move away from deficits or barriers within HIV clinics to a focus on assets and services that can be harnessed to improve the flow through hypertension care cascade to ultimately improve patient-level outcomes among PLWH [29, 30]. This study used the PEN-3 cultural model, an asset-based framework, as a guide with in-depth interviews conducted among key stakeholders involved with HIV services in Akwa Ibom, Nigeria.

Study findings include three core themes that may advance the adoption and long-term sustainability of task-sharing strategies for hypertension among PLWH. These themes include: 1) extending the continuity of care for PLWH; 2) empowering health care providers; and 3) enhancing existing workflows, staff motivations, and stakeholder engagement to complement the recommendations outlined in the Nigerian government task-sharing policies for health care systems. Ideally, efforts to integrate hypertension care within existing HIV service delivery should ensure that they are maximally aligned with existing service delivery for PLWH to meet their needs and reduce the potential for stigma while adhering to the local context in which care is currently being provided. Some settings and cadre of health care workers already provide hypertension services for PLHW. If this is the case, further training and re-training, alongside supervision and open communication, may help ensure that the task-sharing strategies align with existing workflows and routine functions of the HIV clinics.

Our study includes the application of the PEN-3 cultural model as a guide to identify assets within existing HIV clinics that can be harnessed to integrate task-sharing strategies for hypertension among PLWH [31]. The PEN-3 model provides a framework for addressing perceptions that matters to key stakeholders, enablers, or resources that can be nurtured or mobilized to address factors likely to influence the implementation process in resource-limited settings. Further, barriers will exist and oftentimes are the sole focus of most interventions focused on addressing the implementation process in many resource-limited settings. However, the model contributes knowledge based on assets that are positive and or/existential that should be nurtured or mobilized in order to promote the adoption and implementation of task-sharing strategies for hypertension among PLWH attending existing HIV clinics in Nigeria [36, 37]. Among all key stakeholders, one such asset is the role of advocacy, whether at the patient, provider, or policymaker level, which may play a role in strengthening TASSH service delivery for PLWH. Most implementation studies rarely address the advocacy strategies necessary for enhancing the implementation process, and much research is needed to capture the minimum necessary steps that may be crucial in strengthening efforts to integrate TASSH within HIV clinics. Stakeholder advocacy was also perceived in this study to be crucial in enhancing the implementation process. Participants noted the importance of involving stakeholders in the implementation planning, as well as fostering meaningful involvement throughout the life cycle of the project. Our prior experience, engaging stakeholders in the formative stages of an effort to manage hypertension among PLWH, generated ideas such as the need to create awareness among PLWH, foster collaboration with state and local government groups as well as faith-based organizations and other community gatekeepers that can be influential with encouraging PLWH with getting the blood pressure measured regularly [12]. Similar findings have been observed in other studies conducted in Ghana, Malawi, and Uganda, all of which noted that stakeholder engagement may help to close the research-to-practice gap that exists when evidence-based interventions are adapted or implemented in new settings [16, 27, 49].

A major strength of our study is the use of the PEN-3 cultural model to identify assets within existing HIV clinics that can be leveraged to enhance the integration of TASSH [31]. This approach differs from typical implementation science data that are often focused on the deficits within resource-limited settings, rather than positive, existential, or unique assets that can be leveraged to optimize care [29, 30, 37]. This is also a critical step in the translational research pipeline, as interventions focused on existing assets are likely to be adopted and sustained over time. Additionally, using the PEN-3 cultural model as an organizing tool helped illuminate salient perceptions, enablers, or resources, nurturing factors within HIV services that can be leveraged for optimal delivery of hypertension care for PLWH. Finally, our use of a purposive sampling strategy with key stakeholders increased the likelihood of generating knowledge on potential assets that will enable us to characterize the implementation context necessary for optimizing the adoption of TASSH. However, a key limitation of the study include the small number of stakeholders and variability in responses by different stakeholder groups, which may limit generalizability to other settings where culture or readiness for change varies. It would be beneficial to more systematically assess the effectiveness of leveraging assets to drive change. These findings could inform which implementation strategies to use to enhance the adoption and sustainability of TASSH within HIV clinics.

## Conclusion

Integration of evidence-based task-sharing strategies for hypertension care is increasingly common in many African settings also dealing with the persistent high burden of HIV. In our qualitative study of in-depth interviews with key stakeholders involved with HIV service delivery in Nigeria, using the PEN-3 cultural model as a guide, we found that extending the continuity of care among PLWH, empowering health care providers while enhancing existing workflow, and staff motivation and stakeholder engagement where potential assets that can be harnessed to optimize the benefits of hypertension care for PLWH. A, greater understanding of how assets work in limited resource settings, including knowing their mechanisms of change [50], will facilitate the development of implementation strategies that improve the adoption and sustainability of TASSH for PLWH attending HIV clinics in Nigeria.

## Supporting information

S1 File In-depth interview guide. (DOCX)

S2 File Consolidated criteria for reporting qualitative research. (PDF)

S3 File Inclusivity in global research questionnaire. (DOCX)

S4 File Minimal data set. (PDF)

## List of abbreviations

TASSH: task-sharing strategies for hypertension care
PLWH: People living with HIV
HIV: Human Immunodeficiency Virus
SSA: Sub-Saharan Africa
NCDs: Non-Communicable Diseases
CHEWs: Community Health Extension Workers
