# Supplementary material for: Assets for integrating task-sharing strategies for hypertension within HIV clinics: Stakeholder’s perspectives using the PEN-3 cultural model
Source: PLoS One. 2024 Jan 2;19(1):e0294595. doi: 10.1371/journal.pone.0294595 (PMC10760724; doi:10.1371/journal.pone.0294595)
Supplement: S1 File — (DOCX) [file pone.0294595.s001.docx]

**Background**

Good day, thank you for accepting to participate in this study. My name is ______ and I am working with the MAP-IT Study. The reason for this interview is to understand ways in which we might improve hypertension care within SIDHAS-supported primary health clinics. Please, keep in mind that there are no right or wrong answers. I am interested in your thoughts and opinions. Also, you do not have to answer any question that you do not want to answer. Please note that any information you share during this interview is confidential and will not be shared with anyone outside the research team. Kindly confirm that you have voluntarily agreed to participate in this interview and have signed the consent form.

This interview will be audio-recorded. The audio-recording is done to make sure that we capture exactly what you have said and not what we remember you saying. Your name or any identifying information about yourself or anyone that you mention during the interview will not be associated with your responses when making a written transcript of the interview. I want to know if it would be okay to continue with the audio recording? Please stop me anytime if you feel we should stop audio taping.

**SOCIODEMOGRAPHIC INFORMATION**

| **No.** | **Question** | **Response Options** | **Code** |
| --- | --- | --- | --- |
| 1. | Participant ID Number | [insert instructions for how to create participant id] | **[_______________]** |
| 2. | Date of interview | DD/MM/YY |  |
| 3. | Interviewer ID number |  | **[_______________]** |
| 4. | Gender | Male | **1** |
|  |  | Female | **2** |
| 5. | Age |  | **[________] years** |
|  |  |  |  |
| 6. | Educational background (Select highest level completed or candidate) | Primary school | **1** |
|  |  | Secondary school | **2** |
|  |  | Technical school | **3** |
|  |  | Bachelor’s degree (or candidate | **4** |
|  |  | Master’s degree (or candidate) | **5** |
|  |  | Doctoral degree (or candidate) | **6** |
| 8. | Professional title |  |  |
| 9. | Position/post |  |  |
| 10. | Years Worked |  | **[_____] years** |
| 11. | Years worked in your Specialty |  | **[_____] years** |

**INTERVIEW GUIDE FOR POLICYMAKERS/MANAGERS**

1. We want to explore this TASSH to see how far it would be able to work in our present system. So, what do you think about TASSH as a strategy for hypertension control?
2. To what extent do you believe TASSH can be carried out in supported PHCs, specifically SIDAS-supported?
3. What would be some benefits to having some nurses provide hypertension care at the primary healthcare level?
4. Do you envisage any challenge or barrier in implementing TASSH? Do you foresee any…?
5. What factors do you think will enhance the successful implementation of this Task sharing, Task strengthening?
6. Based on your experiences, we would like to understand your thoughts on how PHCs might respond to the introduction of these new strategies, that’s TASSH, for managing new, for managing patients at the PHC level. What do you think?
7. What key stakeholders do you think should be involved in the decision-making process or implementation of TASSH?
8. How do you think the opinion of key stakeholders will influence TASSH at the facility level?
9. To what extent do you think these stakeholders you have mentioned have the authority to carry out the expected change that we expect to see, that is the change on the Task sharing idea?
10. To what extent do you think that these key stakeholders will be able to influence the implementation of TASSH in the facility?
11. What other priorities do you think might interfere with implementing TASSH?
12. To what extent do physicians or nurses see task sharing of hypertension care? that is do they see it as appropriate or do they consider it inappropriate?
13. To what extent do key stakeholders at the PHC usually collaborate across professions, disciplines, units, specialties, and departments? Do they collaborate effectively?
14. To what extent do SIDAS-Supported PHCs value open communication, dialog, and staff inputs?
15. Is there any other additional thought or insight you may want to mention?
16. Thank you so much for your time. Is there any other thing you may want to add?

**INTERVIEW GUIDE-PATIENT ADVOCATES**

1. What do you know about high blood pressure?
2. Do you think controlling high blood pressure is important for people living with HIV?
3. What are those things that can go wrong if a PLHIV with hypertension does not have their hypertension controlled?
4. What experiences have you had with having a healthcare provider talk to you about, or treat you for high blood pressure?
5. Would you want to receive other types of health services like treatment for high blood pressure at the same place where you receive HIV services?
6. What kind of health services would you like to receive through the HIV Clinic?
7. How confident are you in nurses’ ability to manage high blood pressure? And when I say nurses, across the spectrum, whether primary, secondary, and tertiary?
8. How willing are you to change your HIV care visit schedule to get other health services such as control of high blood pressure?
9. Do you think there are barriers that would make some persons not get willing to visit the facility more frequently to address issues relating to high blood pressure?
10. What do you think will be the challenges to the implementation of TASSH in primary health care centers?
11. Thank you very much for giving me the opportunity to interview you. Is there any other thing you will like to add?

**INTERVIEW GUIDE-HEALTHCARE PROVIDERS**

1. What do you think about Task Shifting for Hypertension Control (TASSH)?
2. Do you think there are benefits to Strengthening Hypertension Control at the level of PHC?
3. Do you think there are barriers to Strengthening Hypertension Control at the level of PHC?
4. Do you believe that PHCs will be able to implement TASSH?
5. Why do you think they can implement it?
6. Which people at your PHC do you think should be involved with the process?
7. What should be the process of introducing TASSH in your PHC? How do you think TASSH should be designed in your PHC?
8. What are the factors that will enhance the implementation of TASSH in your facility?
9. Are there other factors that you think will enhance the implementation?
10. What key stakeholders will influence the implementation of TASSH in your clinic?
11. At the level of the PHC, would focal persons across different units, play a role in the success or in implementation of TASSH in your facility?
12. How would they play a role in the implementation of TASSH?
13. In your opinion, what influences the process of changing care strategies at your clinic?
14. From your experience at your PHC, can you kindly share how willing people are to change practices or care strategies?
15. To what extent do the key stakeholder usually collaborate across professions or units? You have focal persons across different units, so, how well do they collaborate to attain change or to effect a particular clinical process?
16. To what extent does your PHC value open communication, dialog, and staff inputs? can you give us instances to reflect on?
17. To what extent do nurses see hypertension control as appropriate or inappropriate to their role?
18. To what extent do you think nurses or community health workers will have the authority or power to carry out the expected task? That is components of TASSH like blood pressure measurement, prescription of medications for high blood pressure, and lifestyle counseling.
19. How do you think TASSH will fit into current priorities in your PHC?
20. To what extent do you feel that you are being supported by your colleagues and/or leaders in your PHC for professional development?
21. Does the government train you for tasks that they expect you to carry out? For example, if you are to commence COVID-19 vaccination, are you trained ahead of it?
22. If you have done a good job, how is it recognized?
23. How do you find out if there are areas where you need to improve?
24. Do you have additional thoughts or insight? Or do you have any questions you want to ask me?

Thank you for your willingness to undertake this interview.
