# Supplementary material for: Assets for integrating task-sharing strategies for hypertension within HIV clinics: Stakeholder’s perspectives using the PEN-3 cultural model
Source: PLoS One. 2024 Jan 2;19(1):e0294595. doi: 10.1371/journal.pone.0294595 (PMC10760724; doi:10.1371/journal.pone.0294595)
Supplement: S4 File — (PDF) [file pone.0294595.s004.pdf]

Excerpts of the transcripts relevant to the study

"I think it will be cool. Because from there, the people have already known your history, and they know the kind of drugs, they know the type of ART you are taking, and whether you are faced with high blood pressure and you will feel very free, because someone that you have been meeting, you have been meeting over years, and you will feel free to, actually say, this is what is happening to me, this is how it is, and the person knows your history already. So, he will know how to address other related issues." PA06

“Once the vital signs are being conducted when you arrive and your blood pressure is continually higher than a hundred and twenty, definitely that’s an indication that hypertension is around the corner, so, they (nurses at the health center) will refer you to further care in comprehensive sites.” PA-04

“It also helps to control stigma and is more confidential to the client if they continue to access their care in one place, because leaving here and going to another place, people will ask questions. They can say things like, “this one is having double chronic diseases”. Stigma will set in.” PA-05

“You will feel very free, because someone that you have been meeting, over the years, is the same person that you can actually say, this is what is happening to me, this is how it is, and the person knows your history already. So, they will know how to address other related health issues.” PA-06

“We (patient advocate) make a referral also from the primary health to comprehensive site, because they (nurses at the site) have been trained and have the ability to carry out new care given the ongoing hypertension problem in the state.” PA-04

“I’m seeing much benefits than barriers or challenges. Benefit number one, the nurses in the community, they are the people that have a good rapport with PLWH. Thus, they are close to the patient and we have the techniques of ensuring that each visits the hospital… We are the one to check on them when they visit… I’m believing that when the workers will be trained from all cadres of nursing and the equipment made available, the job (TASSH) will be successful." HCP04

"My primary health clinic is able to implement task-sharing because it is something we already do with the issue of blood pressure…we already measure this. It is a practice that has already been on the ground in several units, like the Maternity Unit, the Family Planning, and even the outpatient department. Now, incorporating it into the system will not be a problem.” HCP04

"we’ll gain much from it because instead of sending patients home, at least people

will be there to take care of them. If the doctors are not there, at least the nurses or other workers can try to provide care." HCP03

“The benefits of task-sharing strategies are to prevent hypertension and also to build the capacity of the nurses and the CHEWs involved in the care. It will also help in the early diagnosis and, the early detection and diagnosis of hypertension as well as prevent complications with hypertension like stroke, kidney disease, heart failure, and sudden death. Currently, it is not the duty of

nurses (at primary health centers) to provide hypertension care, however, we now have the opportunity (through such programs like TASSH) of being trained to manage PLWH with hypertension." HCP06

"At this facility, the nurses are very few, very few nurses; and we divide them into shifts. By the time you put them into shifts, some shifts may result in no nurse at all…Because of that, we also try to shift some duties to the Community Health Extension Workers (CHEWs) that are with us, and I think that is the aspect of the task-sharing strategies that would be beneficial to us, so that they (CHEWs) can also be able to take blood, measure blood pressure and then record accordingly…because we don't have enough nurses to do the work. But we have few nurses here, we have CHEWs here, we have health attendants, and all other workers. And I think in the issue of task shifting and sharing, everyone should be involved because it's a team work." HCP07

"what will enhance the implementation of task-sharing strategies for hypertension care is having more staff that are trained and have the capacity to handle the program… when patients appear before them, and the card of these patients is before them, and since they are being incorporated in the TASSH program, they will now see the question on patient's care, "Has this person's blood pressure been checked?" If it's not checked, okay, let me know the patient's blood pressure. So, it will help. And if they discover the patient is hypertensive, they will make sure that the patient gets immediate care… as a result, there should be proper training and re-training of staff." HCP06

"The first important thing is we must have a good blood pressure monitor. Because without having a monitor, you would not know when the blood

pressure is high…Given the necessary equipment…; the necessary tools, and the necessary update of knowledge, they (staff) would be able to carry out the components of the TASSH program." HCP07

"Like in this facility, we have heads of units' meeting; the focal persons would meet and, because we work as a team, you cannot just say I am doing family planning, and then, family planning cannot stand alone, immunization cannot stand. So, all the focal persons, we meet, and then we interact and make sure the team moves together. And, if you don't do that, you know, somebody will stand alone and the program will not work…We have open dialog during these meetings…Because you know, without communication, you cannot succeed." PM02

"You need to also factor in how to motivate them. When we're talking about motivating them, it might not just be financial motivation. No. From time to time, appreciate them and tell them, "O, you are useful. You are relevant. You're contributing to the success of these things. Everybody, I think at that level, people want to be appreciated". PM03

"Well, we collaborate actions, because we have monthly meetings where we review our activities. Once a month, we pick a particular case, discuss it among ourselves and we suggest the way forward, and what should be done. That is how we collaborate within ourselves".PM01

"We have a shortfall in the number of healthcare workers in the state, and so many programs are coming in at the same time. We are doing COVID-19 vaccination right now. Then, immunization plus days are also coming in. Net replacement campaign is also coming in. A lot of things" PM02

"The operational base, that controls the other smaller PHCs under them, might have better facilities, might have well-trained nurses, and some of those cases. So, you need to carefully select, you know, the facilities, you need to carefully select based on where you have the right infrastructure for you to work with." PM03

" The stakeholders would definitely have to include all people expected to be involved. So that's the level of the government, from the community, people that are running the program. The state government provides the enabling environment, allowing this program to be fully incorporated formally as part of the things that are offered on a routine basis. Okay, So they advocate that level. Policy making level can be incorporated cause of this, just so that they provide the enabling environments and a *Strategic health Plan will be needed for reflecting to what extent this program fits with priorities. " PM04*
